# Shape evolution of ooids: a geometric model

**DOI:** 10.1038/s41598-018-19152-0

**Published:** 2018-01-29

**Authors:** András A. Sipos, Gábor Domokos, Douglas J. Jerolmack

**Affiliations:** 1 Department Mechanics, Materials and Structures, Budapest University of Technology and Economics, Budapest, 1111 Hungary; 2 MTA-BME Morphodynamics Research Group, Hungarian Academy of Sciences – Budapest University of Technology and Economics, Budapest, 1111 Hungary; 3 Department Earth and Environmental Science, University of Pennsylvania, Philadelphia, Pennsylvania 19104 USA

## Abstract

Striking shapes in nature have been documented to result from chemical precipitation — such as terraced hot springs and stromatolites — which often proceeds via surface-normal growth. Another studied class of objects is those whose shape evolves by physical abrasion — the primary example being river and beach pebbles — which results in shape-dependent surface erosion. While shapes may evolve in a self-similar manner, in neither growth nor erosion can a surface remain invariant. Here we investigate a rare and beautiful geophysical problem that combines both of these processes; the shape evolution of carbonate particles known as ooids. We hypothesize that mineral precipitation, and erosion due to wave-current transport, compete to give rise to novel and invariant geometric forms. We show that a planar (2D) mathematical model built on this premise predicts time-invariant (equilibrium) shapes that result from a balance between precipitation and abrasion. These model results produce nontrivial shapes that are consistent with mature ooids found in nature.

## Introduction

**Figure 1:**
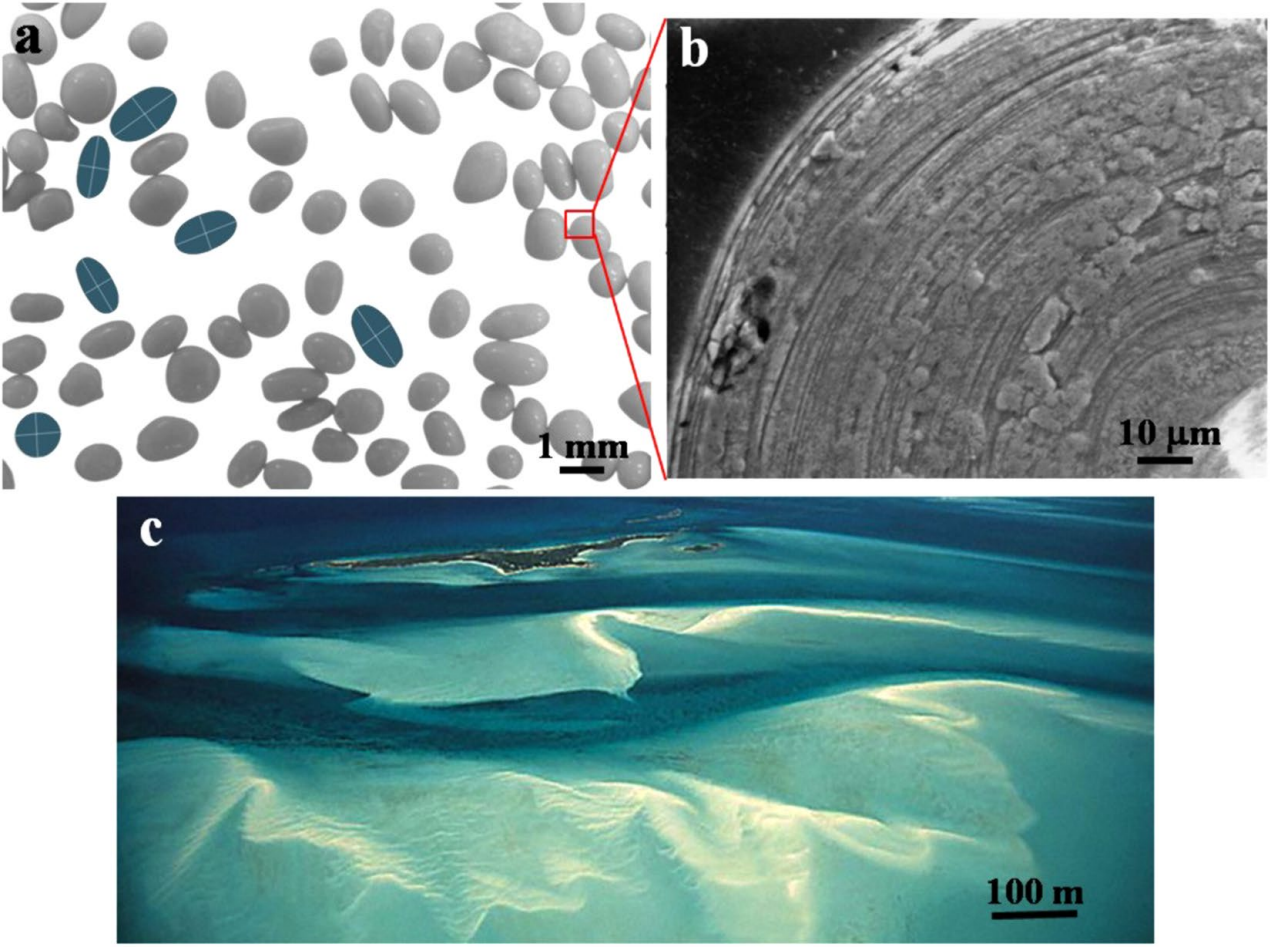
Ooids and their environment, with scale bars indicated for each image. (**a**) Ooids sampled from a sand shoal in Joulters Keys, Bahamas. Some example shapes are highlighted in blue. White lines show the approximate axes of reflectional symmetry. (**b**) Close-up cross section of an ancient ooid showing concentric layers. This ooid is not in (**a**), but is shown relative to (**a**) for context and scale. Image credit: Wikipedia, Creative Commons License. (**c**) Ooid sand shoals in the Bahamas; image from SEPM Strata Website^36^. Reprinted by permission of the SEPM whose permission is required for further use.

Ooids are rounded, sand-sized particles of calcium carbonate that typically form by mineral precipitation in warm and shallow coastal waters. Their transport by waves and currents gives rise to spectacular shoals and white sand beaches, for example in the Bahamas^1,2^ (Fig. 1). Because ooids grow under a restricted range of conditions, they are increasingly being investigated for their potential to record environments of the geologic past^3–5^. A remarkable aspect of ooids is that–like trees–they record their own growth history, as accretion of carbonate occurs in concentric layers (Fig. 1). Unlike tree rings, however, the formation of concentric ooid layers is poorly understood^6–8^.

Although aspects of ooid formation remain enigmatic, there is general agreement among modeling, laboratory and field studies on the qualitative picture. In the absence of collisions with other particles, isolated ooids form spheres with little to no layering^6,7^. This may occur in quiescent environments^9^, or when particles are small enough to be suspended by wave action^6^. If growth is restricted by neighboring particles, ooids can become non-spherical and eventually merge together^10^. The mechanism of growth is (possibly biologically-mediated) surface precipitation of calcium carbonate under super-saturated conditions^11,12^. The presence of ooid-sand waves in energetic environments shows that these particles are also transported as bed load^1,2^ – i.e., by rolling, sliding and hopping along the sediment bed – a process that is known to result in abrasion^13,14^. Importantly, ooids transported in bed load often exhibit non-spherical shapes and concentric layering. An additional relevant observation is that layer thickness tends to decrease from the center outwards^15^, suggesting an increase in abrasion rate (relative to precipitation rate) as ooids grow.

Patterns resulting from either growth by chemical precipitation^16,17^, or erosion by physical abrasion^13,18,19^, are well studied. To our knowledge, however, the shape evolution of objects under the action of both has not been examined. Moreover, a systematic study of the shape of ooids, and its evolution recorded in their concentric layers, is lacking. Recent work has made substantial progress on a related aspect of this problem, however;^20^ have demonstrated that the equilibrium *size* of ooids is determined by a balance between growth by chemical precipitation and erosion by abrasion. Here we seek to construct a geometric model that builds on this result, that encapsulates the basic processes of ooid growth and erosion, and that is motivated by and compared to ooid shapes.

## Physical picture

For a particle immersed in a fluid with uniform solute concentration, mineral precipitation is known to result in surface-normal growth^16^. In the absence of abrasion this growth process produces spherical shapes^7^. Abrasion under bed-load transport involves two distinct processes: collisions, which drive erosion toward spherical shapes, and friction, which causes flattened and elongated shapes^21^. For abrasion to occur, particle momentum must be large enough to overcome viscous dissipation. This condition is determined by a critical Stokes number, beyond which abrasion is proportional to particle mass^22^. This implies that abrasion is insignificant for nascent ooids, but becomes increasingly important as ooids grow–consistent with the onset of layering and deviations from surface-normal growth that are observed for ooids above a critical size^6^.

It is uncertain as to whether ooids alternate between periods of growth and abrasion, or if both occur simultaneously. To allow a continuous description of ooid evolution we will assume the latter; even in the case of the former, this assumption should remain valid so long as shape evolution encompasses many growth and abrasion events. From the above picture, one might naively expect that at some size the competing processes of growth and erosion balance each other. This intuition was recently confirmed experimentally by^20^, who clearly demonstrated that ooid size reflects a dynamic equilibrium between rapid precipitation and abrasion rates. Whether this also implies the existence of *invariant shapes* is a more delicate question: shape fluctuation constrained by some conserved scalar quantity (e.g. linear size, perimeter, surface area, energy or mass) has been broadly studied in subjects ranging from flexible membranes to pulsating stars^23–25^ and those results indicate that constant size does not necessarily imply constant shape as well. Here we show that a reasonable mathematical model based on standard surface evolution equations predicts an affirmative answer. A few key additional observations, from the literature and our own work, help to motivate and constrain the development of a model for ooid shape evolution.

1. Ooids found in the same location tend to have roughly similar maximal size^2,20^ and similar shape^1,2^.
2. Ooid shapes range from spherical to elongated pill structures, and these shapes have been proposed to be related to transport environment^10^.
3. Ooid shapes tend to be rotationally symmetric around their longest diameter (major axis) (Fig. 1)^6^.
4. Ooid contours (planar projections of ooids) have two orthogonal axes of symmetry (Fig. 1).

The above observations apply only to *unconstrained* ooids where neither growth nor abrasion was prevented by contact with other ooid particles. Below we develop a minimal model for unconstrained ooid shape evolution. The model is based on the listed four observations; it produces invariant and nontrivial shapes, and it is consistent with the known processes governing ooid formation.

## Results

### Model development

Although ooids are three dimensional objects, they exhibit (approximate) rotational symmetry around the major axis; this observation is supported by published cross sections of ooid particles in the literature (e.g.^6^). Thus the development of a 2D model, aimed at capturing the evolution of the meridian curve (contour), appears to be a plausible first step. We acknowledge that this dimension-reduction is far from trivial, and therefore we provide a critical assessment of the 2D assumption for each step of model development.

#### Abrasion

The geometric theory of abrasion goes back to Aristotle^26^, while the modern theory of individual-particle abrasion operating with partial differential equations (PDEs) appears to start with the work of Rayleigh^27–29^. The first PDE model developed on physical grounds is due to Firey^19^ who considered repeated collisions of an arbitrary convex shape with an infinite plane. Firey’s equation for collisional abrasion can be formulated for a smooth, convex body by expressing the speed *v* in the direction of the inward normal at an arbitrary surface point as

1 $$v={c}_{1}\kappa ,$$

where *κ* is the Gaussian curvature of the particle and *c*_1_ is a constant. Firey’s model (1) can also be interpreted for planar, convex curves Γ: in this case *κ* is the scalar curvature. Firey’s theory was developed further by Bloore^18^ by considering collisions not only with planes, but with objects of finite size, resulting in a generalization of eq. (1) to

2 $$v=1+2b\mu +{c}_{1}\kappa ,$$

where *μ* is the mean curvature, *κ* is the Gaussian curvature, and *b* and *c*_1_ may be constants or integrals (depending on the geophysical interpretation). The planar version of (2) can be written as

3 $$v=1+{c}_{1}\kappa $$

where again *κ* is the scalar curvature at an arbitrary point on the surface of the particle. While there are various geophysical interpretations for (3), one possible interpretation is to regard the abrading environment as (statistically) constant. In this case *c*_1_ appears as a time-independent constant in eq. (3). For the case of convex, rotationally symmetric bodies with moderate elongation, the 2D simplification (3) produces a moderate error compared to (2): among prolate ellipsoids the maximal deviation in the normal speed between the 2D and 3D models is less than 10% provided that the aspect ratio is smaller than 3:1. Since most observed ooids are below this aspect ratio, and we are primarily interested in qualitative features of shape evolution, we use the planar (2D) model to study the evolution of the contour. Ooids are typically well sorted^1,2^, and it has been shown^21^ that collisions among like-sized grains result in abrasion that is qualitatively well approximated by eq. (1) in the sense that all shapes converge to the sphere. Accordingly, we implement eq. (1) to model collisional abrasion.

**Figure 2:**
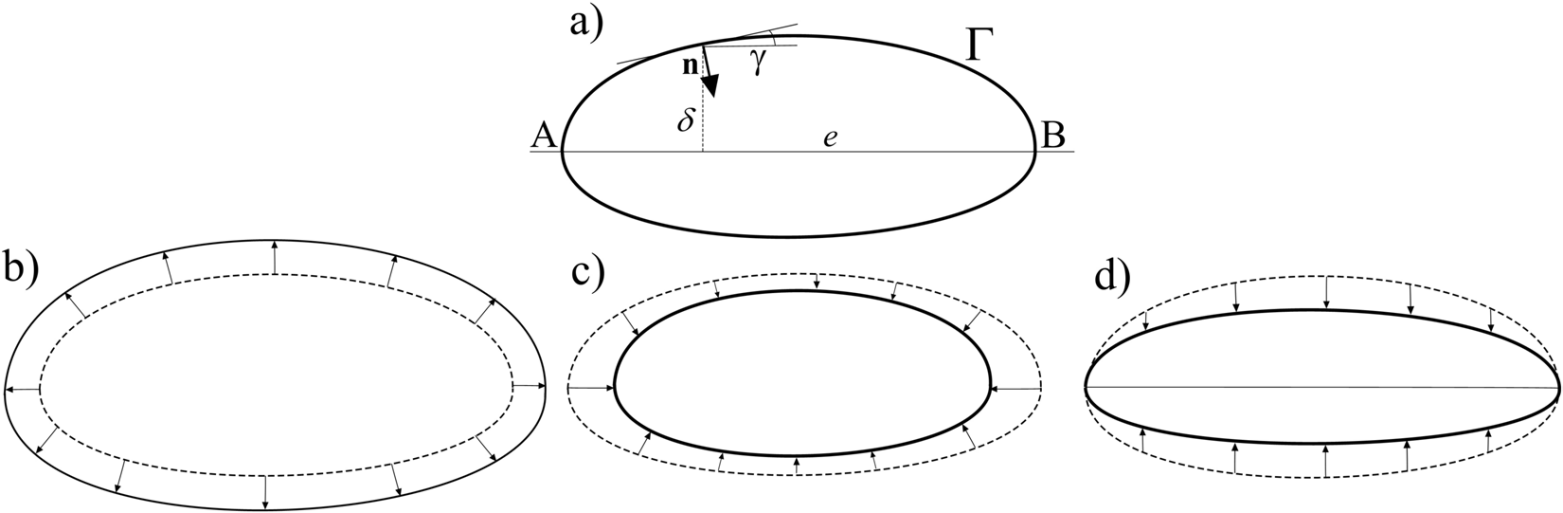
Notations and qualitative features of contour evolution. (**a**) The closed, convex curve Γ with maximal diameter *e*. (**b**) The effect of the constant (growth) term (i.e. first term in (5)) on Γ. All shapes converge to the (infinitely large) circle. (**c**) The effect of the collisional abrasion term on Γ. All shapes converge to the (infinitely small) circle^19^. (**d**) The effect of the frictional abrasion term on Γ. All shapes converge to ‘needles’.

Collisions account only partly for the shape evolution of sedimentary particles; frictional abrasion also plays a key role. Unlike collisions, friction does not have a broadly accepted geometric model. Nevertheless, in^21^ a set of axioms for such models was listed and some specific models have also been investigated. The key idea of frictional abrasion is that–unlike collisional abrasion–it depends not only on the local properties (e.g. curvature) of the surface but also on global ones. In simple terms, flat shapes tend to become flatter under sliding friction, and thin shapes tend to become thinner under rolling friction. Here we implement the simplest possible friction model, orthogonal affinity, consistent not only with the axioms in^21^ but also with the above-described intuitive picture. As we aim to describe shape evolution of rotationally symmetric particles around their largest diameter, orthogonal affinity is applied on the cross section of the particle. In this planar case, sliding and rolling are not distinguished and this model describes the effect of friction as an affine shrinking of the curve orthogonal to its largest diameter *e*. In the PDE model this can be formulated as

4 $$v={c}_{2}\delta \,\cos \,\gamma ,$$

where *δ* is the distance of the investigated point from *e*, *γ* is the angle of the tangent line at the given point with respect to *e* (cf. Fig. 2), and *c*_2_ is a time-independent constant.

#### Growth

Growth phenomena with strictly local rules are commonly described by the Kardar-Parisi-Zhang (KPZ) equation^30^. Again, appealing to rotational symmetry and mild elongation of ooid shapes, as an approximation we use the planar version of the KPZ equation. Similar to the heat equation, the KPZ equation is most often described in fixed, external Cartesian coordinates where its connection to the Bloore equation is hard to realize. However, the KPZ equation also has an intrinsic, invariant formalism developed in^31^ and^32^, and by adopting this approach the planar version can be written as

5 $$v=-1-c\kappa -\chi \mathrm{.}$$

As we can see, (5) is essentially identical to the planar Bloore model given in eq. (3), with an added stochastic term *χ*; if we restrict ourselves to deterministic models then the two equations are identical, with opposite sign. This formulation not only shows the connection between the two equations, but it also provides an intuitive physical interpretation: the first (constant) term corresponds to *area-driven* growth, while the second term corresponds to *curvature-driven* growth. The observed spherical growth pattern of ooids under pure chemical growth^7^ is strong evidence that the first term in the KPZ equation (5) has to be adopted in our growth model, while the second (curvature-driven) term can be neglected.

#### Combined ooid shape evolution model

We consider the simplest PDE model for simultaneous abrasion and growth in two dimensions for a closed, planar curve Γ with no self-intersections. We assume that Γ has a unique maximal diameter *e*. For modeling abrasion, we adopt Firey’s curvature-driven theory given in eq. (1) and the simplest affine friction model given in eq. (4) satisfying the axioms in^21^.

For modeling growth, we consider only the constant *v* = −1 term in the KPZ equation. When adding the growth and abrasion terms we also consider the fact that abrasion is proportional to mass, which in the planar model is represented by the area *A* enclosed by Γ. We do not explicitly treat the threshold Stokes number for the onset of abrasion, but some aspect of this effect is captured in that abrasion is negligible for small mass. Based on these considerations we arrive at the non-local PDE model

6 $$v={c}_{3}(-1+A({c}_{1}\kappa +{c}_{2}\delta \,\cos \,\gamma ))\mathrm{.}$$

The two parameters *c*_1_ and *c*_2_ set the weights of wave-induced collisions and sliding/rolling, respectively, *relative* to growth by precipitation (Fig. 2); they may be scaled in various manners. The shape evolution or *geometry* of the particle is determined entirely by *c*_1_ and *c*_2_, which do not contain any description of rate or timescale. Parameter *c*_3_ is introduced as a kind of *rate constant*, in order to scale the speed of shape evolution; its value is expected to vary with environmental setting depending on chemical and physical conditions. At the moment there are no data available for the shape evolution of ooids in order to test our *local* evolution equation (6). Recent experiments, however, have documented the evolution of volume under growth (${\dot{V}}_{p}$) and abrasion (${\dot{V}}_{a}$)^20^. The net volume growth rate can be described by a *global* model, $\dot{V}=-{\dot{V}}_{p}+{\dot{V}}_{a}$, where clearly an equilibrium volume is reached when abrasion balances precipitation. The simplest global model for volumetric abrasion rate that is consistent with data is a linear relation ${\dot{V}}_{a}\sim V$^33^; a comparable (though slightly more complicated) model was found to describe the experimental evolution of ooid volume well^20^. Our new *local* model (eq. 6) predicts volume evolution of ooids that is exactly consistent with predictions from the *global* linear-abrasion rate model^33^, for the case where *c*_2_ = 0. For *c*_2_ > 0 the friction term appears as an additional non-linear (quadratic) term for abrasion, that may be considered to provide a higher-order approximation at the global scale. A detailed derivation of the equivalence of the global and local models is provided in the Supplementary Material.

For the remainder of this paper we aim to examine novel predictions of ooid shape evolution (rather than volume); without loss of generality we fix *c*_3_ = 1 for simplicity.

### Application

Physical intuition suggests that the combined action of growth and abrasion may result in time-invariant shapes. Indeed, it can be shown rigorously that equation (6) has smooth steady-state solutions, characterized by *v* ≡ 0. Here we describe a comprehensive interpretation of the analytical results, verify them numerically and apply the theory to ooid cross section images. The invariant solutions have rather appealing properties which have been verified rigorously; for our current purpose we merely list them:

1. Ellipses are not invariant solutions, i.e. they do not satisfy the *v* ≡ 0 condition for eq. (6) (see the explanation in the Supplementary Material).
2. Any smooth invariant solution must possess *D*_2_ symmetry, i.e. it must have two orthogonal axes of reflection symmetry.
3. *c*_1_ and *c*_2_ uniquely determine a smooth invariant solution.

**Figure 3:**
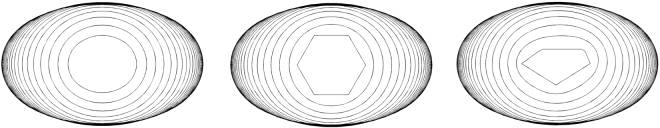
Invariant shapes as global attractors: shape evolution under eq. (6) with parameters *c*_1_ = 0.3, *c*_2_ = 2.0, starting from radically different initial conditions. Observe that regardless of the latter, the asymptotic invariant shape is uniquely determined by the equation and the two parameters. Note also that the concentric isochrons become more closely spaced outward from the center, consistent with observations of ooid layers.

We used the level set method^34,35^, to numerically compute the evolution of curves under eq. (6). Our computations confirm all three listed properties of the postulated invariant shapes. Moreover, we find that for any given *c*_1_, *c*_2_ the invariant shape is also a global, asymptotic attractor (cf. Fig. 3).

**Figure 4:**
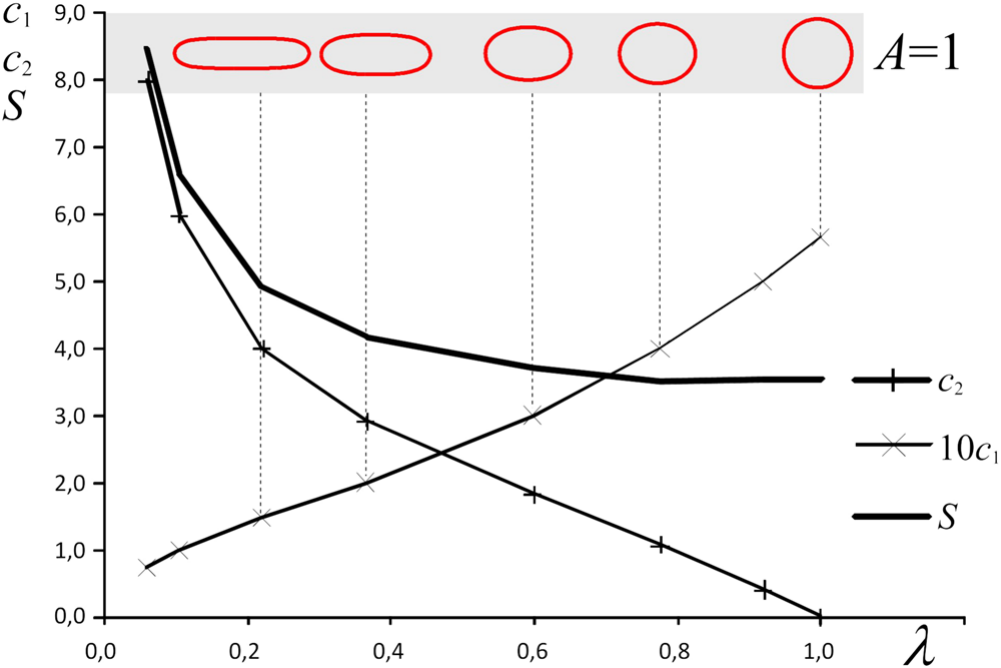
Variation of the parameters *c*_1_ and *c*_2_ and the perimeter *S* versus the aspect ratio *λ* for equilibrium shapes. Observe that invariant shapes with unit area form a one-parameter family as *λ* is varied.

Let us denote the length of the longest diameter by *a*, the length of the smallest diameter by *b* and their ratio by *λ* = *a*/*b*. Since invariant shapes are fully determined by two parameters *c*_1_, *c*_2_, if we fix the area *A* then we arrive at a one-parameter family of shapes which can be conveniently parametrized by *λ*. This is illustrated in Fig. 4, which can also be used to graphically determine the model parameters for measured values of *λ* — under the assumption that ooid shape evolution achieved equilibrium. If, beyond measuring the long and short axes *a* and *b* we are also able to measure the curvatures at their endpoints, then the model parameters *c*_1_, *c*_2_ can be expressed by simple closed formulae.

## Discussion

With analytical results confirmed by numerical simulations, we can now make some observations. The parameters *c*_1_, *c*_2_ represent the physical environment; in particular, the respective strength of collisional and frictional abrasion relative to growth by precipitation. Our model predicts that in the same environment we should find identical shapes and sizes *so long as all particles have reached equilibrium* and the growth has been unconstrained. Natural settings may contain mature and immature ooids, while sediment transport by waves and currents may influence observed size distributions. Nonetheless, the prediction of invariant shapes is consistent with Observation (1) in the Introduction. In principle, our model also allows one to separate mature and immature ooids based on their shape alone: as shown in the Results Section, mature shapes form a one-parameter family which can be characterized by the axis ratio *λ*. This implies that the curvature *κ* at the endpoints of the contour can be given as *κ*(*λ*) on mature ooids. If the measured shapes show a different *κ* vs *λ* relationship then we may conclude that the evolution process did not reach equilibrium yet. It would be interesting to observe how this property correlates with the maximal size of ooids.

**Figure 5:**
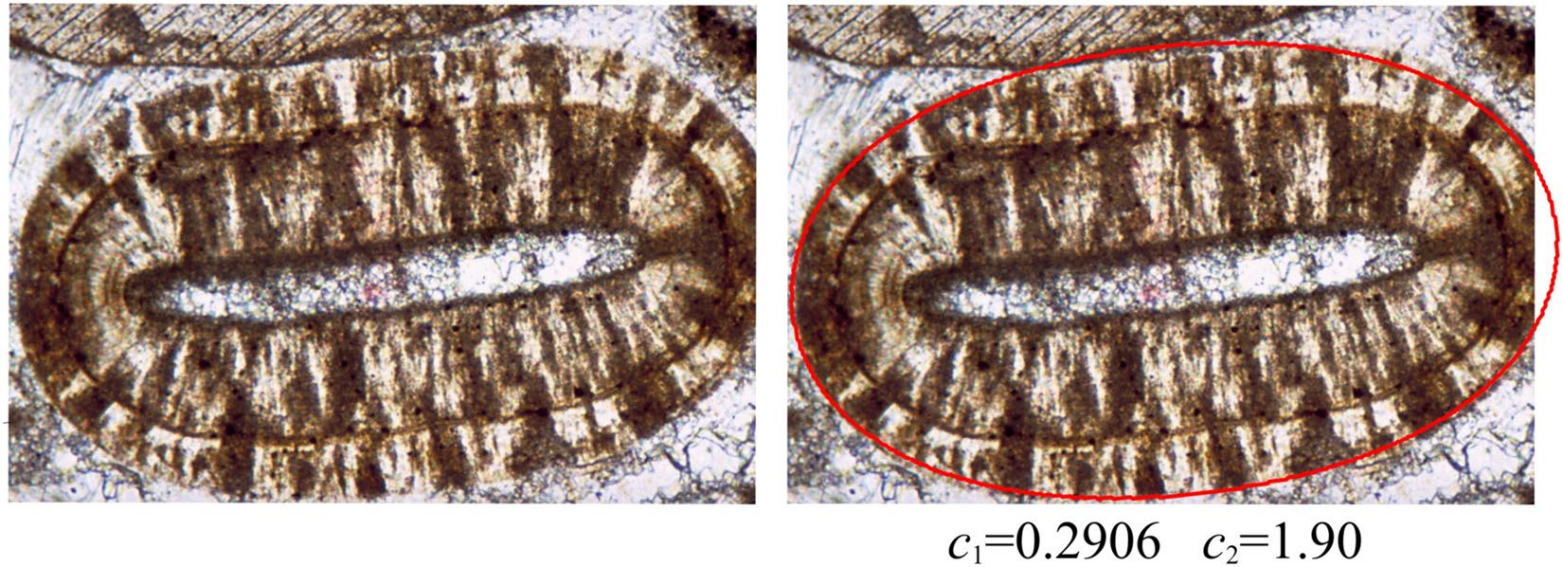
An elongated ooid shape and the fitted invariant shape of the model (*c*_1_ = 0.9206, *c*_2_ = 1.90). Image used for the figure is from^37^, p226, AAPG©2003. Reprinted by permission of the AAPG whose permission is required for further use.

**Figure 6:**
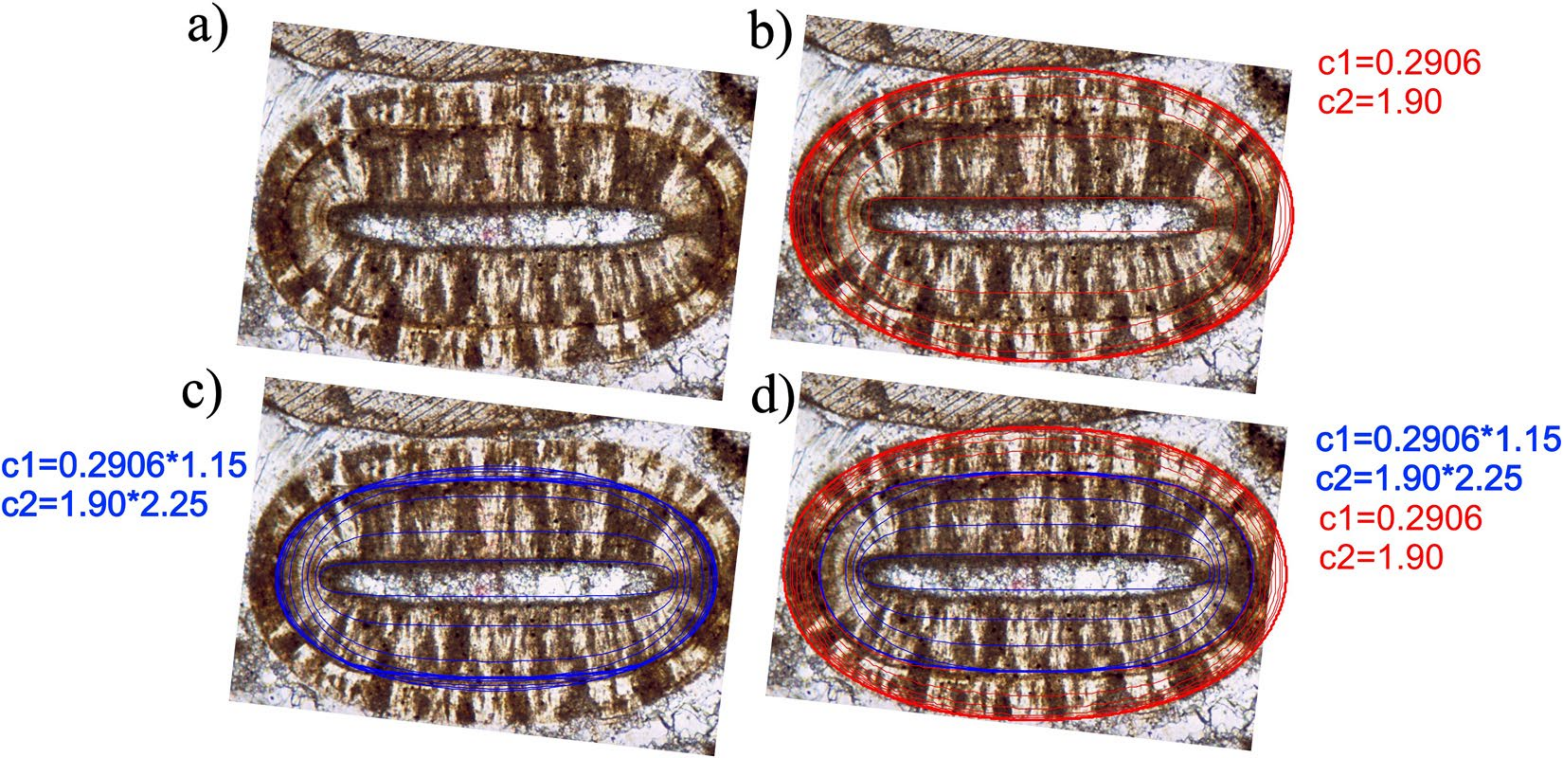
A two-phase model to explain intermediate shapes. (**a**) An ooid with a significant intermediate contour. (**b**) Model parameters that predict the final shape to be invariant do not capture the intermediate contour. (**c**) With different model parameters the intermediate contour is close to an invariant shape (observe that the maximal lateral extent of the shape is observable before the invariant shape occurs). (**d**) The two phase model composed from the fits of panels (b) and (c). Evolution to the final state was initiated at the intermediate contour. Image used for the figure is from^37^, p226, AAPG©2003. Reprinted by permission of the AAPG whose permission is required for further use.

While it is not (yet) our goal to achieve a quantitative match, the model appears to capture the non-elliptical geometry of ooids reasonably well. Figure 5 shows an example ooid contour with model fit (*c*_1_ = 0.9206, *c*_2_ = 1.90). As we can see, there seems to be a discontinuity in the evolution of this ooid, as reflected by the intermediate contours. This discontinuity may indicate a change in the environment. In Fig. 6 we illustrate two different fitted invariant shapes; one models the evolution between the core and the intermediate contour, and the other corresponds to the evolution between the intermediate contour and the final shape of the grain. Obviously, an invariant intermediate contour should be associated with lower speed for growth. This is indeed the case in our simulations, as the values of *c*_1_ and *c*_2_ are higher for the fit of the intermediate curve compared to the ones associated with the final shape. Apparently, the two-phase model provides a reasonable approximation also for the intermediate curves.

The complexity of our model is not sufficient to aim for quantitative agreement between model predictions and physical shapes, however, there are several immediate natural generalizations of the model which allow for more free shape parameters. One such generalization is to consider not only the second, but also the first (constant) term in eq. (3). The next step would be to consider the 3D versions of all listed models. These generalizations introduce new model parameters which could be used to match measured contours to higher accuracy. Nonetheless, predictions from the planar model could already be tested with new laboratory experiments that tune growth and erosion parameters independently. In particular, we predict that ooid growth under a constant environment saturates due to a balance between surface precipitation and abrasion, and produces an invariant shape that reflects the relative magnitudes of these processes. If verified, the model could be used to invert ooid shape for formative environment, aiding interpretation of the geologic record.

## Electronic supplementary material

Supplementary Material
