# Author Correction: Shape evolution of ooids: a geometric model

**DOI:** 10.1038/s41598-018-24105-8

**Published:** 2018-04-13

**Authors:** András A. Sipos, Gábor Domokos, Douglas J. Jerolmack

**Affiliations:** 1 Department Mechanics, Materials and Structures, Budapest University of Technology and Economics, Budapest, 1111 Hungary; 2 MTA-BME Morphodynamics Research Group, Hungarian Academy of Sciences – Budapest University of Technology and Economics, Budapest, 1111 Hungary; 3 Department Earth and Environmental Science, University of Pennsylvania, Philadelphia, Pennsylvania 19104 USA

Correction to: *Scientific Reports* 10.1038/s41598-018-19152-0, published online 29 January 2018

This Article contains an error in Equation 5.

*v* = −1 − *cκ* − *χ*

should read:

*v* = −1 + *cκ* − *χ*

As a result, this Article contains errors in the Results section under subheading ‘Growth’.

“As we can see, (5) is essentially identical to the planar Bloore model given in eq. (3), with an added stochastic term *χ*; if we restrict ourselves to deterministic models then the two equations are identical, with opposite sign. This formulation not only shows the connection between the two equations, but it also provides an intuitive physical interpretation: the first (constant) term corresponds to *area-driven* growth, while the second term corresponds to *curvature-driven* growth.”

should read:

“As we can see, (5) is essentially identical to the planar Bloore model given in eq. (3), with an added stochastic term *χ*; if we restrict ourselves to deterministic models then the two equations are identical, with opposite sign for the constant term. This formulation not only shows the connection between the two equations, but it also provides an intuitive physical interpretation: the first (constant) term corresponds to *area-driven* growth, while the second term corresponds to *curvature-driven diffusion*.”

