# Supplementary material for: Shape evolution of ooids: a geometric model
Source: Sci Rep. 2018 Jan 29;8:1758. doi: 10.1038/s41598-018-19152-0 (PMC5789073; doi:10.1038/s41598-018-19152-0)
Supplement: Supplementary file 1 — Supplementary Material [file 41598_2018_19152_MOESM1_ESM.pdf]

Supplementary Material for the paper Shape evolution of ooids: a geometric model

András A. Sipos[1,2,*], Gábor Domokos[1,2], and Douglas J. Jerolmack[3]

[1]Dept. Mechanics, Materials and Structures, Budapest University of Technology and Economics, Budapest 1111, Hungary
[2]MTA-BME Morphodynamics Research Group, Hungarian Academy of Sciences – Budapest University of Technology and Economics, Budapest, 1111, Hungary
[3]Dept. Earth and Environmental Science, University of Pennsylvania, Philadelphia, Pennsylvania, 19104, USA
[*]siposa@eik.bme.hu

ABSTRACT

In Appendix 1 we show that the $c_2 = 0$ version of our model is exactly consistent with the model presented in (Trower et al. 2017) if for global abrasion we adopt the model by Bathurst (1975). We define how the constants (i.e. specific rates) in the two models correspond to each other. We also show that the friction term in our model appears to be the natural extension of the model in (Trower et al. 2017), as it yields to a second-order term in volume reduction with respect to mass.
In Appendix 2 we prove that ellipses are not invariant shapes of the model presented in the paper.

Appendix 1: Correspondence between the global model in Trower et. al. (2017) and our local model

The model presented in Tower et al.(2017)[1] is based on the work by Bathurst (1975)[2]. The latter states that the precipitation rate is proportional to particle's surface area S and abrasion is proportional to the volume V of the grain and thus it is also proportional to the mass of the grain. (The latter term is modified in Trower et al (2017) to accommodate nonlinearity for larger grains, however, here we consider the Bathurst (1975).) This is a global model, the net change of the volume of the grain is given by eq. (1) in[1]:

$$\dot{V}_{net} = \dot{V}_p - f\dot{V}_a, \tag{1}$$

where upper dot refers to derivation respect to time, $\dot{V}_p$ and $\dot{V}_a$ denote the rate of change of volume due to precipitation and abrasion, respectively and f denotes the relative time spent in transport. Without loss of generality we fix $f = 1$. In the Bathurst (1975) model the rates can be expressed as

$$\begin{aligned}\dot{V}_p &= c_{01}S,\\ \dot{V}_a &= c_{02}m = c_{02}\rho V,\end{aligned} \tag{2}$$

where c_{01}, c_{02} are constants, m, S and ρ are the mass, surface area and density of the ooid, respectively. In the two-dimensional (planar) case we can write

$$\begin{aligned}\dot{A}_p &= c_{01}\Gamma,\\ \dot{A}_a &= c_{02}m = c_{02}\rho A,\end{aligned} \tag{3}$$

so the planar Bathurst model reads:

$$\dot{A}_{net} = c_{01}\Gamma - c_{02}\dot{V}_a, \tag{4}$$

where A is the area of the (planar) grain and Γ is the perimeter of the grain.

The local model, operating with local speeds, v_{net}, v_p and v_a associated with net change, growth and abrasion of the surface in the normal direction, respectively, aims not only to determine the changes in volume (area) but also to determine the changes in shape. We call a local model *consistent* with a global model if it predicts the same mass evolution as the global model (and beyond that, it also predicts some kind of shape evolution). Below we show that our local model

$$v_{net} = v_p - v_a = c_3\left(-1 + c_1 A\kappa + c_2 A\delta\cos\gamma\right) \tag{5}$$

is in 2 dimensions consistent with a natural generalization of (4).

In the first step we note that the integrals of the local speeds v_{net}, v_p and v_a over the perimeter Γ of the grain (surface area S of the grain in the 3D case) equal the global rates $\dot{A}_{net}$, $\dot{A}_p$, $\dot{A}_a$:

$$\begin{aligned} \dot{A}_{net} &= \int_\Gamma v_{net} ds, \\ \dot{A}_p &= \int_\Gamma v_p ds, \\ \dot{A}_a &= \int_\Gamma v_a ds. \end{aligned} \tag{6}$$

Integrating equation (5) along the arc length of the curve, using the Gauss-Bonnet theorem for the curvature term and the geometric interpretation of the friction term (linear affinity with factor c_2c_3) we obtain:

$$\dot{A}_{net} = \int_\Gamma v_{net} ds = \int_\Gamma c_3 \left(-1 + c_1 A\kappa + c_2 A\delta \cos\gamma\right) ds = c_3\Gamma - 2\pi c_1 c_3 A - c_2 c_3 A^2, \tag{7}$$

and we can see that with the substitution $c_1 = (\rho c_{02})/(2\pi c_{01})$, $c_2 = 0$, $c_3 = c_{01}$, equation (7) is identical to equation (4). This implies that in the absence of friction ($c_2 = 0$) our local model is consistent with the Bathurst model.

Observe that our frictional term predicts, on the global level, an abrasion rate proportional to the second power of the area. If we consider the abrasion rate to be a general function of the mass (area) then our friction term captures the second term of the Taylor polynomial. We also remark that the above correspondence between the local and global model remain valid also for the 3D case; however, we do not consider that problem here.

Appendix 2: Ellipses are not invariant shapes

Since the friction term corresponds to orthogonal affinity for $c_2 > 0$, one might naively expect ellipses as invariant shapes. We show that this is not the case. First let us investigate the $c_2 = 0$ case when

$$-1 + c_1 A\kappa = 0 \tag{8}$$

holds, thus $\kappa \equiv$ const for any point along the curve. It implies *circles* are the only invariant shapes at $c_2 = 0$. For the general case ($c_2 \neq 0$) we use proof by contradiction. We assume an ellipse with $a > b$ semi-axes is in steady state. We parametrize the (in this case elliptic) arc between points A and P in the well-known way

$$x(\phi) = a\cos\phi, \qquad y(\phi) = b\sin\phi, \tag{9}$$

where $0 \leq \phi \leq \pi/2$. The curvature of the parametrically-defined curve is given by

$$\kappa(\phi) = \frac{|x'y'' - x''y'|}{(x'^2 + y'^2)^{3/2}} = \frac{ab}{(a^2\sin^2\phi + b^2\cos^2\phi)^{3/2}}, \tag{10}$$

with $()'$ and $()''$ denoting the first and second derivatives with respect to ϕ. Considering that the area of an ellipse is $A = ab\pi$ and $\cos\gamma = \cos(\arctan(y'/x'))$, the expression in eq. (5) can be written as

$$-1 + c_1 \frac{a^2b^2\pi}{(b^2\cos^2\phi + a^2\sin^2\phi)^{3/2}} + c_2 \frac{ab^2\pi\sin\phi}{\sqrt{1 + \frac{b^2\cos^2\phi}{a^2\sin^2\phi}}} = 0. \tag{11}$$

At the endpoint of the major axis, $\phi = 0$ and $c_2 = 0$. With this result in hand, after simplification we obtain

$$c_1 = \frac{b}{a^2\pi}. \tag{12}$$

In a similar manner we substitute $\phi = \pi/2$ and, using the value for c_1 from eq. (12), we obtain:

$$c_2 = \frac{a^3 - b^3}{a^4b^2\pi}. \tag{13}$$

Finally, we take a third value of ϕ to demonstrate that, with the derived constants c_1 and c_2, the equation is not satisfied. For example, after substitution of c_1, c_2 and $\phi = \pi/4$ into eq. (11) we obtain

$$-1 + \frac{b^3}{(0.5a^2 + 0.5b^2)^{3/2}} + \frac{\sqrt{2}}{2} \frac{a^3 - b^3}{a^3\sqrt{1 + b^2/a^2}} \neq 0. \tag{14}$$

The left side of this equation is not identically zero; truncating around $a = b$, one can show that $a = b$ makes it vanish. We found that, among ellipses, only the circle is a possible steady-state candidate; in that case, c_2 must vanish.

References

1. E.J. Trower, M.P. Lamb and W.W. Fischer. Experimental evidence that ooid size reflects a dynamic equilibrium between rapid precipitation and abrasion rates. *Earth and Planetary Science Letters*, 468:112–118, 2017.
2. R.G.C. Bathurst. Carbonate Sediments and Their Diagenesis. *Elsevier Publishing Co., Amsterdam*, 1975.
